# The role of TDP-43 propagation in neurodegenerative diseases: integrating insights from clinical and experimental studies

**DOI:** 10.1038/s12276-020-00513-7

**Published:** 2020-10-13

**Authors:** Myungjin Jo, Shinrye Lee, Yu-Mi Jeon, Seyeon Kim, Younghwi Kwon, Hyung-Jun Kim

**Affiliations:** 1 Dementia Research Group, Korea Brain Research Institute (KBRI), Daegu, 41062 South Korea; 2 Department of Brain & Cognitive Sciences, DGIST, Daegu, 42988 South Korea

**Keywords:** Neurodegenerative diseases, Neurodegeneration

## Abstract

TAR DNA-binding protein 43 (TDP-43) is a highly conserved nuclear RNA/DNA-binding protein involved in the regulation of RNA processing. The accumulation of TDP-43 aggregates in the central nervous system is a common feature of many neurodegenerative diseases, such as amyotrophic lateral sclerosis (ALS), frontotemporal dementia (FTD), Alzheimer’s disease (AD), and limbic predominant age-related TDP-43 encephalopathy (LATE). Accumulating evidence suggests that prion-like spreading of aberrant protein aggregates composed of tau, amyloid-β, and α-synuclein is involved in the progression of neurodegenerative diseases such as AD and PD. Similar to those of prion-like proteins, pathological aggregates of TDP-43 can be transferred from cell-to-cell in a seed-dependent and self-templating manner. Here, we review clinical and experimental studies supporting the prion-like spreading of misfolded TDP-43 and discuss the molecular mechanisms underlying the propagation of these pathological aggregated proteins. The idea that misfolded TDP-43 spreads in a prion-like manner between cells may guide novel therapeutic strategies for TDP-43-associated neurodegenerative diseases.

## Neurodegenerative disorders: Spread of misfolded protein aggregates

Further research is needed to determine how an aggregate-forming protein common to several neurodegenerative disorders propagates throughout the brain. Many neurodegenerative conditions involve aggregates created by ‘prion-like’ proteins, misfolded proteins that can confer their abnormal structure on neighboring healthy proteins, resulting in aggregates which spread rather like an infection. Hyung-Jun Kim at the Korea Brain Research Institute in Daegu, South Korea, and co-workers reviewed current understanding of the transactive response DNA-binding protein 43 (TDP-43), an aggregate-forming protein implicated in disorders such as Alzheimer’s disease and frontotemporal dementia. Growing evidence suggests that TDP-43 may spread in a prion-like fashion. TDP-43 is implicated in the onset of Alzheimer’s, and the spread of misfolded TDP-43 aggregates is closely tied to disease severity. More research is needed into how TDP-43 propagates in different tissues and central nervous system cells.

## Introduction

Amyotrophic lateral sclerosis (ALS), Parkinson’s disease (PD), frontotemporal dementia (FTD), Alzheimer’s disease (AD), and limbic predominant age-related TDP-43 encephalopathy (LATE) are common neurodegenerative diseases with an increased prevalence in aging societies. Although the clinical symptoms of these diseases are different, they share a similar pathological feature. The formation and accumulation of pathological inclusions composed of abnormal aggregated proteins in affected tissues is one of the hallmarks of these neurodegenerative diseases^1,2^. Each of these diseases is associated with misfolding of specific proteins. For example, the main proteins comprising pathological deposits in AD are amyloid-β and tau^3–5^. α-Synuclein is the major component protein of pathological deposits in PD^6,7^. Furthermore, TAR DNA-binding protein 43 (TDP-43) aggregates are frequently observed in multiple diseases, such ALS, FTD, AD, and LATE^8–10^.

TDP-43 is a ubiquitous protein that is encoded by the *TARDBP* gene and belongs to the heterogeneous nuclear ribonucleoprotein (hnRNP) family. In normal cells, TDP-43 is mainly present in the nucleus and plays important roles in RNA regulation, such as transcriptional regulation, alternative splicing, and mRNA stabilization^11–13^. Under pathological conditions, cleavage, hyperphosphorylation and ubiquitination of TDP-43 can occur^14–16^. These posttranslational modifications lead to cytoplasmic accumulation and aggregation of TDP-43. In particular, phosphorylation of TDP-43 at serine 403/404 and 409/410 (p-TDP-43) can result in the pathological inclusions observed in TDP-43 proteinopathies^16–18^.

Recently, several studies have been conducted on the propagation of these misfolded pathological proteins based on clinical evidence^19–21^. These so-called ‘prion-like’ proteins have a common domain that can induce misfolding and self-aggregation^22–24^. It has also been revealed that such pathological proteins can play roles as seeds that can be propagated to other nearby cells in various ways to serve as templates for creating new aggregates. Extensive research has shown that amyloid-β, tau, and α-synuclein aggregates can induce native protein misfolding and cell-to-cell transmission both in vitro and in vivo^25–31^. Similarly, while evidence of a prion-like mechanism of amyloid-β, tau, and α-synuclein aggregation has been reported for over a decade, the mechanism of TDP-43 propagation is relatively unknown.

In this review, we focus on evidence of spreading TDP-43 pathology in several neurodegenerative diseases and summarize the published experimental studies supporting cell-to-cell propagation of TDP-43 both in vitro and in vivo.

## Properties of TDP-43: genetics, structure, and localization

**Fig. 1:**
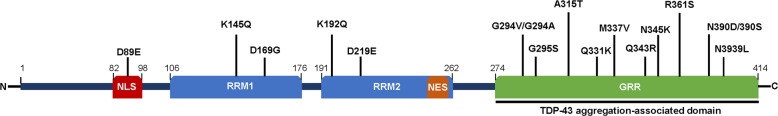
Schematic representation of the structure and aggregation-associated mutations of TDP-43. The TDP-43 protein contains a nuclear localization signal (NLS), 2 RNA-recognition motifs (RRM1 and RRM2), a nuclear export sequence (NES), and a glycine-rich region (GRR). Numerous disease-associated TDP-43 mutations have been identified in the GRR. Most disease-associated TDP-43 missense mutations have been identified to accelerate TDP-43 aggregation.

TDP-43 is composed of an N-terminal domain (NTD; residues 1–103), two RNA recognition motifs (RRM1 and RRM2; residues 104–200 and residues 191–262), and a C-terminal domain (CTD; residues 274–413). The NTD region contains a ubiquitin-like fold with one α-helix and six β-sheets and promotes TDP-43 self-oligomerization in a concentration-dependent manner^32,33^. Mutations of the nuclear localization sequence (NLS) in the NTD are the cause of cytoplasmic localization and aggregation of TDP-43^34^ (Fig. 1). TDP-43 contains two RRM domains that comprise five β-strands and two α-helices. These regions have been shown to bind UG/TG-rich single-stranded or double-stranded DNA/RNA to perform various functions in transcriptional repression, pre-mRNA alternative splicing, and translational regulation^35,36^. The C-terminus of TDP-43 is essential for solubility and cellular localization of the TDP-43 protein and regulates protein–protein interactions^37^. This domain is rich in glycine, glutamine, and asparagine. These unusual sequences resemble those of the prion-like domain^22,23^. Prion-like domains are low-complexity sequences enriched in uncharged polar amino acids (asparagine, glutamine, and tyrosine) and glycine, and their sequence properties are similar to those of yeast prion proteins. The prion-like domain plays a key role in regulating the solubility and folding of proteins. Proteins containing prion-like domains undergo phase separation into membraneless, spherical compartments^38^. The liquid–liquid phase separation of TDP-43 is influenced by both hydrophilic and hydrophobic residues. The presence of mutations or aberrant posttranslational modification leads to the formation of irreversible aggregation via liquid–solid phase separation^39^. Many mutations in the TDP-43 gene have been found to be associated with ALS and FTLD, and most disease-associated mutations are located within the C-terminal domain^40,41^. In particular, in several models, these mutations in the C-terminal domain of TDP-43 can promote the intrinsic aggregation of TDP-43. The expression of TDP-43 mutations, including Q331K, M337V, Q343R, N345K, R361S, and N390D, has been shown to increase aggregation and cell toxicity in yeast cells^41^, and other disease-associated mutations, such as G294A, Q331K, M337V, Q343R, N390D, and N390S, enhance protein aggregation when expressed in SH-SY5Y cells^42^. In a *Drosophila* model, the expression of mutant TDP-43 A315T has been shown to increase protein aggregation and neurotoxicity^43^. In addition, various peptides bearing pathogenic TDP-43 mutations, such as G294V, G294A, and G295S, have been found to form twisted amyloid-like fibers^44^. Therefore, the CTD domain of TDP-43 is the most relevant region of the protein for its aggregation.

Normally, TDP-43 is predominantly localized in the nucleus, but it also shuttles between the nucleus and the cytoplasm to carry out diverse cellular functions. The level and localization of TDP-43 is finely regulated via a negative-feedback mechanism^45^. However, under stress conditions such as heat shock, oxidative stress and arsenite exposure, the cytoplasmic transfer of nuclear TDP-43 increases, and cytoplasmic TDP-43 accumulates to form stress granules (SGs) with several other proteins and RNAs^46–49^. When stress dissipates, SGs containing TDP-43 disassemble, and TDP-43 released from SGs translocates into the nucleus^50^. However, chronic stress evokes prolonged SG formation, which leads to persistent accumulation of cytoplasmic TDP-43 aggregates.

In addition to dysregulation of SG formation, dysfunction of the nuclear pore complex is also related to cytoplasmic mislocalization and aggregation of TDP-43. Several studies have reported that the cytoplasm–nucleus gradient of Ran, the major regulator of nuclear localization of TDP-43, is reduced by the expression of (G_4_C_2_)_30_ RNA. (G_4_C_2_)_30_ RNA also disrupts the structure of the nuclear membrane^51^. Moreover, other nuclear membrane proteins, such as Nup62 and Kpnb1, are associated with the cytoplasmic accumulation of TDP-43^52^. In addition, cytoplasmic aggregates of TDP-43 can directly induce the disruption of nucleocytoplasmic transport and nuclear pore complexes^53,54^. TDP-43 aggregates induce the mislocalization and aggregation of nucleoporins and transport factors. TDP-43-induced impairment of the nuclear pore complex accelerates cytoplasmic mislocalization and accumulation of TDP-43, subsequently contributing to neuronal dysfunction and toxicity.

Some TDP-43 missense mutations can enhance the mislocalization of TDP-43. TDP-43 has two signal sequences, an NES and an NLS, which regulate the cellular localization of TDP-43 in the cytoplasm and nucleus. An ALS-linked A90V mutation in the NLS facilitates the cytoplasmic aggregation of TDP-43^55^. Several C-terminal mutations in TDP-43, such as G294V, A315T, M337V, A382T, and G376D, promote cytoplasmic mislocalization and aggregation through mechanisms that have not yet been clarified^34,56,57^. Certain ALS-related TDP-43 mutants, such as G348C, A315T, and Q343R, generate larger SGs than wild-type TDP-43 as well as abnormal SGs^46,58^.

## Clinical evidence of TDP-43 propagation in neurodegenerative diseases

### Alzheimer’s disease and limbic predominant age-related TDP-43 encephalopathy

AD is a progressive neurodegenerative disease and the most common type of dementia. Amyloid-β or tau deposition is generally regarded as a major cause of the pathogenesis of AD. However, recent studies have revealed that TDP-43 is closely related to the onset and development of AD^59–62^. TDP-43 pathology is observed in between 20% and 50% of AD patients and in 75% of patients with severe AD^63–65^. In AD patients, TDP-43 pathology may begin in the amygdala and spread to the area of the cortex that regulates memory^21,63^. In a study on a large cohort of AD patients, p-TDP-43 (marker of TDP-43 aggregates) immunoreactivity in the amygdala was detected in a higher percentage of AD patients than normal subjects. p-TDP-43 deposition progresses to the entorhinal cortex and subiculum, which are next to the occipitotemporal cortex and dentate gyrus of the hippocampus, followed by the temporal cortex, substantia nigra, midbrain, inferior temporal cortex, basal ganglia, and middle frontal cortex^21,61,63,66^. TDP-43 pathology stages 1–5 are associated with impairment of episodic, semantic, and working memory, perceptual speed and visuospatial ability are related to TDP-43 accumulation in AD^61,67^.

Previous studies have suggested that the propagation mechanism of TDP-43 in AD involves direct cell-to-cell transmission^68–70^ or distant cell-to-cell transmission^19^. Recently, a consensus working group published new terminology and diagnostic criteria for undefined neuropathology entities, LATE, and limbic predominant age-related TDP-43 encephalopathy neuropathological change (LATE-NC). The research group examined whether some patients diagnosed with AD do not exhibit accumulation of amyloid-β protein through a postmortem study. This study revealed that accumulation of TDP-43 protein but no accumulation of amyloid-β protein was observed in patients with AD-like symptoms. Excessive accumulation of TDP-43 leads to cognitive disorders, and 20–50% of patients aged 80 or older have sufficient levels of TDP-43 aggregates to induce cognitive disorders. In LATE-NC, which differs from FTLD-TDP in the region affected, the distribution of TDP-43 pathology is relatively limited. TDP-43 aggregates are found only in the amygdala in the first stage of LATE and are present in the amygdala and hippocampus in the second stage. TDP-43 inclusions are found in the amygdala, hippocampus, and middle frontal gyrus in the third stage^9,71^. TDP-43 pathology in LATE patients is associated with the same mechanism of cell-to-cell transmission described above.

### Frontotemporal lobar degeneration (FTLD)

FTLD is a progressive and fatal neurodegenerative disease characterized by deficits in behavior or language skills associated with degeneration of the frontal and anterior temporal lobes. FTLD patients have a characteristic histopathology with cytoplasmic inclusions containing aggregated TDP-43 or tau protein in neurons and glial cells. FTLD is classified into different pathological subtypes, including FTLD with tau-positive inclusions (FTLD-tau), FTLD with FUS-positive inclusions (FTLD-FUS), and FTLD with TDP-43 and ubiquitin inclusions (FTLD-TDP). Although TDP-43 mutations are associated with a very low percentage of FTLD cases, TDP-43-positive cytoplasmic inclusions are present in up to 50% of FTLD patients^16,72,73^. In particular, behavioral variant FTD (bvFTD) patients have widespread and severe TDP-43 pathology. The spread of p-TDP-43 pathology was divided into four stages based on the findings of a clinical cohort study. In the first stage, p-TDP-43 inclusions are widespread in the basal and anterior portions of the prefrontal neocortex (orbital gyri, gyrus rectus, and inferior frontal gyrus) and amygdala. In stage 2, an increased p-TDP-43 burden is observed in the anteromedial area, superior and middle temporal gyri, striatum, and medial and lateral portions of the thalamus. In more advanced cases, a third stage with involvement of the motor cortex, neocortical areas, and spinal cord anterior horn is observed. In stage 4, the p-TDP-43 inclusion burden spreads to the occipital neocortex region, the visual processing center of the brain^20,74,75^. p-TDP-43 lesions in FTD have been associated with various behavior, language, and functional abilities and are closely connected to the prefrontal cortex, limbic structures (amygdala and hippocampus), and striatal regions^20,76,77^. Moreover, the spreading pattern of FTD is similar to that of ALS, possibly indicating a common molecular mechanism among p-TDP-43 proteinopathies^20^. p-TDP-43 pathology spreads to distinct parts of the brain through the major axonal pathway via cell-to-cell transmission as described above.

### Amyotrophic lateral sclerosis

ALS (also known as Lou Gehrig’s disease) is the most common motor neuron degenerative disease and is characterized by progressive degeneration of both upper and lower motor neurons. Less than 10% of ALS cases are familial ALS (fALS), and ~4% of fALS cases are caused by mutations in the gene encoding *TARDBP*. Although a small proportion of sporadic ALS and fALS cases are associated with TDP-43 mutations, TDP-43 pathology can be observed in more than 90% of ALS patients^14,15,78^.

The initial symptoms of ALS can be fairly diverse in different people and can be found in specific parts of the body. The symptoms also tend to be asymmetrical. As the disease progresses, the symptoms generally spread from one side to both sides of the body. In addition, the progression rate of ALS can be quite variable from one person to another. The severity of motor neuron loss is also related to the site of disease onset. Although not all people with ALS experience the same symptoms or the same sequence or progression pattern, progressive muscle weakness and paralysis are universally experienced^79–81^. These clinical features may evidence for the cell-to-cell propagation of TDP-43.

According to studies by Brettschneider, J. et al. (2013) and Braak, H. et al. (2013), ALS can be divided into the following four stages. In stage 1, p-TDP-43 inclusions mainly occur in the projection neurons of the agranular motor cortex and in the somatomotor neurons of the brainstem and spinal cord. In stage 2, p-TDP-43 aggregates are observed in the prefrontal cortex, reticular formation, precerebellar nuclei of the brainstem, and parvocellular portions of the red nucleus. In stage 3, p-TDP-43 pathology develops in the prefrontal cortex, striatum, and basal ganglia. In stage 4, p-TDP-43 pathology extensively progresses into the anteromedial areas of the temporal lobe and entorhinal cortex and in the hippocampal and dentate fascia^19,82^. Clinical observations of ALS patients have shown that ALS progression is characterized by an increase in p-TDP-43 lesions as well as the degeneration of motor neurons^82^. Several studies have revealed that p-TDP-43 aggregates are sequentially propagated in axons of somatomotor neurons via axonal transport^19,82,83^. Axonal transport is therefore thought to have an essential role in p-TDP-43 pathology in ALS, possibly through the corticospinal tract. However, the molecular mechanisms of p-TDP-43 pathology must be elucidated in future studies.

## Experimental models of TDP-43 propagation

### In vitro studies using conditioned medium

Conditioned medium experiments have been used to investigate the possible transmission of cell-derived TDP-43. In a study published by Feiler et al.^84^, TDP-43 was fused to the N-terminal or C-terminal half of luciferase constructs named TDP-L1 and TDP-L2, and luciferase activity was detected if TDP-L1 and TDP-L2 interacted. Conditioned medium from HEK293 cells transiently transfected with TDP-43 fused to luciferase or TDP-L1 and TDP-L2 was collected after 72 h and centrifuged to eliminate floating cells and cell debris. Native HEK293 cells were cultured in conditioned media for 72 h, and luciferase activity was measured in recipient cells after extensive washing. The results showed intracellular uptake of TDP-43 aggregates from the medium and were confirmed in primary mouse cortical neurons using an rAAV6.2 viral vector harboring TDP-L1 and TDP-L2. In addition, to determine whether TDP-43 is transmitted by microvesicles/exosomes (MVEs), HEK293 cells were transfected with Myc-tagged TDP-43. Then, MVEs were collected after 72 h. Western blot analysis confirmed the presence of Myc-tagged TDP-43 in the MVEs. In addition, researchers performed microfluidic culture system experiments to examine the possible uptake and transmission of TDP-43 by axon terminals. This experimental technique allowed the culture of neuronal cell bodies fluidically isolated from their axon terminals. Conditioned medium from HEK293 cells transfected with TDP-Luc was added to the axon terminals for 5 days. Then, luciferase activity was detected in lysates of primary cortical neuronal soma from the opposite chamber. The hypothesis that intracellular TDP-43 aggregates can be released through exosomes was also confirmed in another study^85^. The presence of TDP-43 in the exosomal fractions of SH-SY5Y cells expressing TDP-43 and treated with/without brain insoluble lysates from ALS patients was detected by immunoblot analysis.

However, in another published study using conditioned medium, researchers were unable to detect TDP-43 aggregates in cells incubated with conditioned medium for 3 days^86^. Similar results have been reported in other papers^87^. These researchers used wild-type TDP-43 and mutant TDP-43 with a dysfunctional nuclear localization signal (ΔNLS) construct and HEK293 cells to obtain conditioned medium. Then, recipient cells, HEK293 cells, and primary mouse spinal cord neurons were incubated with conditioned medium for 20 h. Immunofluorescence staining and immunoblotting of the recipient cells showed no signs of propagation of TDP-43.

These discrepant results from experiments using conditioned media may have resulted from differences in the TDP-43 constructs, cell type, and incubation time. Therefore, more research on the propagation of TDP-43 released by cells is required.

### In vitro studies using a coculture system

It is also possible that TDP-43 undergoes cell-to-cell transfer through contact between cells. To confirm whether TDP-43 aggregates can be propagated between cells, contact coculture experiments were performed. Human neuroblastoma SH-SY5Y cells were transfected with a DsRed or TDP-43 vector^85^. After 3 days, DsRed-transfected cells and TDP-43-transfected cells were mixed at a 1:1 ratio and grown for an additional 3 days. Then, the cells were stained with an anti-pTDP-43 S409/410 antibody and observed by confocal laser microscopy. TDP-43 aggregates were observed in the cytoplasm of DsRed-expressing cells. The proportion of DsRed and phosphorylated TDP-43 double-positive cells was calculated to be 2.9%. The presence of phosphorylated TDP-43 aggregates in cells expressing DsRed indicated that p-TDP-43 aggregates propagated to adjacent cells that originally did not have TDP-43 aggregates. Similar results have been confirmed in other papers^86^. In another study^84^, in a coculture system, TDP-43 was fused to the N-terminal or C-terminal half of VenusYFP (constructs named TDP-V1 and TDP-V2), and VenusYFP fluorescence was observable by microscopy if TDP-V1 and TDP-V2 were oligomerized. Separate cultures of HEK293 cells were transfected with either TDP-V1 or TDP-V2. After 48 h, each transfected cell line was trypsinized and washed, and the cells were coplated at a 1:1 ratio and cocultured for an additional 48 h. After 2 days, cytoplasmic VenusYFP fluorescence was detected in the cocultured cells. This observation indicated that TDP-V1 and TDP-V2 were transferred between cells. In addition to experiments involving fluorescence image analysis methods using microscopy, studies evaluating TDP-43 propagation through single-cell analysis based on flow cytometry have recently been conducted^88,89^. Before flow cytometry, SH-SY5Y ‘recipient’ cells were stably transfected with GFP, and SH-SY5Y ‘donor’ cells were transfected with HA-tagged TDP-43 using the CMV lentiviral system. Equal amounts of recipient cells and donor cells were cocultured for 3 days. The percentage of GFP+ and HA+ (Cy5) cells was measured by flow cytometry, and there was a significant 1% increase in the percentage of double-positive cells 3 days after coculture compared with time point zero^88^. Furthermore, in a recently published paper, NSC-34 cells were transiently transfected with either wild-type TDP-43-tdTomato or mutant TDP-43 G294A-tGFP. Transfected cells were cocultured at a 1:1 ratio, and 24 h later, they were analyzed using flow cytometry. Cell-to-cell transfer was quantified as the percentage of tGFP and tdTomato double-positive cells. More than 10% of the transfected cells contained both tGFP and tdTomato^89^.

### In vitro studies using brain lysates or CSF from patients

Several studies have been carried out to investigate whether insoluble TDP-43 derived from the brain tissues or cerebrospinal fluid (CSF) of ALS or FTLD-TDP patients as a seed can form intracellular inclusions. Prion-like proteins propagated from surrounding cells can play a role as seeds and can induce protein aggregation in healthy cells. SH-SY5Y cells were transiently transfected with HA-tagged TDP-43 and transduced with sarkosyl-insoluble faction containing TDP-43 aggregates prepared from the brain tissues of ALS or FTLD-TDP patients. After 2 days of transduction, intracellular TDP-43 aggregation in SH-SY5Y cells was observed by immunoblot analysis and immunofluorescence analysis. In addition, this seeding activity of insoluble TDP-43 from the brains of neurodegenerative disease patients was stable against detergents, heat, and proteolytic digestion, and cell-to-cell transmission ability was maintained^85^. Two other studies using brain tissue from ALS patients also showed a seeding effect of insoluble TDP-43. Cortex-derived ALS patient samples, but not cerebellum-derived samples, induce TDP-43 oligomerization in primary cultured neurons^84^. Western blotting of HEK293 cells cotransfected with p-TDP-43 extracts from the brains of ALS patients and the full-length TDP-43 construct revealed pathological p-TDP-43 bands. However, HEK293 cells transfected with only brain extracts did not exhibit TDP-43 pathology^86^. Recently, a research group developed a new simple method for pathological TDP-43 extraction, SarkoSpin^90^. By coupling the SarkoSpin method and mass spectrometry, these researchers identified a subset of insoluble proteins beyond TDP-43 in the FTLD subtype. To determine whether there are differences in cell toxicity between types of FTLD-TDP, a cell toxicity assay was performed on mouse primary cortical neurons using SarkoSpin pellets from patients with different disease subtypes. Cell viability was significantly lower after inoculation with FTLD-TDP-A extracts than after inoculation with control or FTLD-TDP-C extracts. Moreover, whether the observed neuronal toxicity is dependent on the aggregation of endogenous TDP-43 was evaluated. p-TDP-43 aggregates extracted from FTLD-TDP-A patients, but not from FTLD-TDP-C patients, were shown to induce endogenous TDP-43 aggregation, potentially via protein seeding.

In addition to experiments using lysates of brain tissue derived from patients, research assessing TDP-43 propagation using CSF from ALS or FTD patients has been conducted^91^. To examine whether TDP-43 aggregates can propagate via CSF, researchers established a CSF cell culture model using U251 cells. U251 cells were inoculated in DMEM containing 30% v/v CSF derived from ALS or FTD patients. After 21 days of CSF treatment, U251 cells showed a decreased rate of growth and morphological changes. The seeding activity of CSF from patients was also confirmed in this culture system. The formation of TDP-43 aggregates and the mislocalization of TDP-43 from the nucleus to the cytoplasm were observed in ALS-FTD-CSF-treated U251 cells. This finding indicates that CSF from ALS and FTD patients contains seeds for inducing TDP-43 aggregation in cells. Moreover, immunofluorescence staining showed that TDP-43 aggregates colocalize with tunneling nanotube (TNT)-like structures in CSF-treated cells. Therefore, propagation of TDP-43 between neighboring cells could be mediated via TNT.

### In vivo studies

As described previously, many experiments have been conducted to show that TDP-43 propagates in a cell-to-cell manner in a cell culture model. However, the evidence for TDP-43 spreading in an in vivo model is not sufficient. In a paper published in 2016, researchers attempted to confirm the results of the in vitro experiments in vivo in a mouse model^92^. The researchers first evaluated whether TDP-43 aggregates are secreted by exosomes in N2a cells. TDP-43 was detected in the exosomal fraction of N2a cells transfected with wild-type human TDP-43, disease-associated mutants (A315T and G348C), or 25 C-terminal constructs. TDP-43 was also detected in exosomes from primary cortical neurons. It has been confirmed that the secretion of exosomes, including the secretion of TDP-43, is controlled by autophagy, the proteasome and protein aggregation. N2a cells expressing hTDP-43 were treated with bafilomycin A1 (an autophagy inhibitor), MG132 (a proteasome inhibitor), or ethacrynic acid (an oxidative stress inducer), and as a result, increased secretion of exosomes was observed. In addition, exosomes from the postmortem temporal cortices of sporadic ALS patients with TDP-43 pathology contained more TDP-43 than exosomes from controls. These results indicate that TDP-43 is secreted with exosomes at higher levels in the brains of ALS patients than in the brains of normal controls. Researchers found that exosomes from ALS patients are internalized by N2a cells or HEK293 cells and that TDP-43 was redistributed in the cytoplasm. To determine whether exosome secretion can buffer TDP-43 aggregates, N2a cells expressing hTDP-43 and transgenic mice expressing genomic fragments encoding mutant TDP-43 A315T were treated with GW4896, an inhibitor of nSMase2 that can reduce exosome secretion. In an in vitro model, treatment with GW4896 reduced exosomal TDP-43 secretion and increased cytoplasmic TDP-43 aggregates. In a transgenic mouse model, GW4896 administration exacerbated abnormal behavioral phenotypes, such as deficits in recognition memory, in TDP-43 A315T mice. Moreover, the number of denervated neuromuscular junctions was increased by GW4896 administration in TDP-43 A315T mice. Therefore, the authors of this paper suggest that inhibition of the exosomal secretion of TDP-43 could reduce the spreading of TDP-43 pathology to other cells, but reducing exosome secretion of TDP-43 as a treatment strategy for ALS might provoke the opposite effect. In another published paper^93^, researchers demonstrated that TDP-43 aggregates in FTLD-TDP spread throughout the brain via cell-to-cell propagation using transgenic mice expressing human TDP-43 (CamKIIa-hTDP-43 NLSm). Brain-derived FTLD-TDP extracts were stereotaxically injected into the neocortex, hippocampus, and thalamus. At 1 month postinjection, p-TDP-43-positive neuronal cytoplasmic inclusions were detected in the neocortex and hippocampus. To further examine the propagation of TDP-43, the authors analyzed the distribution of p-TDP-43 in CamKIIa-hTDP-43 NLSm transgenic mice injected with FTLD-TDP extracts over time. There was an increase in TDP-43 pathology in wider areas of the contralateral cortex over time, indicating the time-dependent spread of TDP-43 pathology to sites distant from the injection site. Researchers have also demonstrated that endogenous TDP-43 acts as a seed and that TDP-43 pathology propagates over time in nontransgenic mice injected with brain-derived FTLD-TDP aggregates, although at a very low rate. These findings indicate that pathophysiological conditions affecting homeostasis and the accumulation of cytoplasmic TDP-43, such as cellular stress, may promote the seeding and propagation of TDP-43 pathology in the brain.

### Molecular mechanism of TDP-43 propagation

**Fig. 2:**
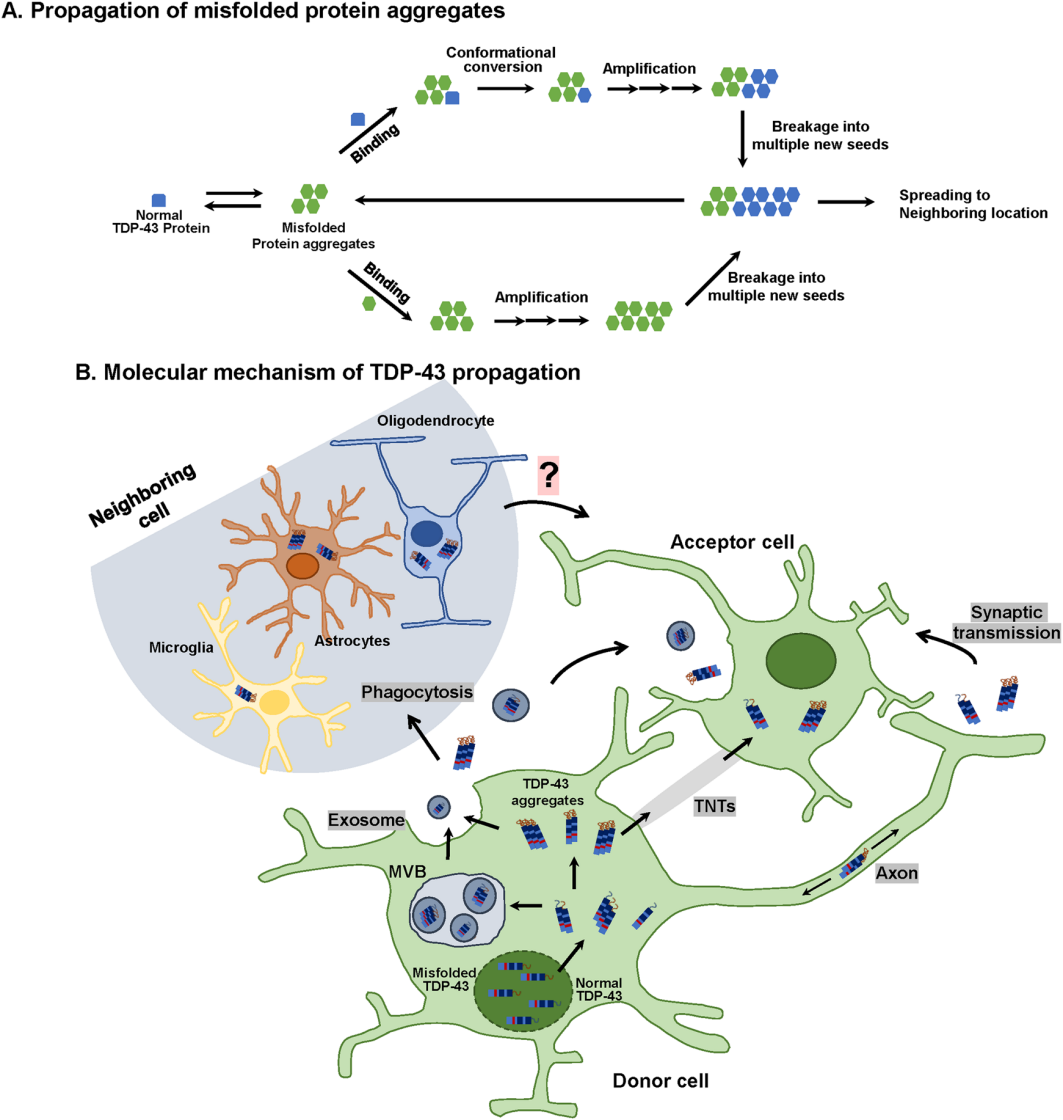
Schematic overview of misfolded TDP-43 propagation in TDP-43 proteinopathies. **a** Proposed mechanism of self-propagation of misfolded TDP-43 in TDP-43 proteinopathies. Misfolded TDP-43 aggregates bind to their normal counterparts and induce the misfolding of bound protein in a template-dependent manner. This process leads to the elongation of misfolded TDP-43 aggregates. Amplification of self-templating amyloid fibrils results from the fragmentation of TDP-43 aggregates, which exposes new ends. **b** Putative mechanism of cell-to-cell spreading of TDP-43 aggregates. TDP-43 aggregates may propagate via exosomes (release from multivesicular bodies (MVBs)), tunneling nanotubes (TNTs), or synaptic transmission (transport from presynaptic to postsynaptic terminals) from donor cells to acceptor cells. Moreover, glial cells (oligodendrocytes, astrocytes, and microglia) can take up TDP-43 aggregates through phagocytosis, after which misfolded TDP-43 is released from glial cells and transmitted to neurons and neighboring glial cells. The neuron-to-glia or glia-to-neuron transfer of TDP-43 has been observed, but its propagation mechanism is not clear.

As previously described, the hypothesis that TDP-43 propagates to neighboring cells through exosomes is controversial. Although the opposite of the expected behavioral results have been observed, it has been shown that the aggregation and propagation of TDP-43 may also be regulated by the exosome pathway in vitro as well as in vivo. In addition, previous studies have revealed that prion proteins^94^ and prion-like proteins, such as amyloid-β^95^, α-synuclein^96^, and tau^97^, propagate via exosomes. Another possible mechanism of TDP-43 protein propagation involves TNTs. TNTs have been reported to play a role in the spread of prion proteins^98^. In experiments using CSF from ALS-FTD patients, it has been confirmed that TDP-43 aggregates that form within one cell might enter the cytoplasm of neighboring cells through a TNT-like structure. Therefore, the mechanisms of other prion-like proteins may be applicable to TDP-43 aggregates (Fig. 2).

## Conclusion

As summarized in this review, it is clear that the propagation of TDP-43 protein occurs in several neurodegenerative diseases. Indeed, accumulating evidence from clinical and basic research (Table 1) indicates that the spreading of misfolded TDP-43 aggregates is closely correlated with the progression and severity of neurodegenerative diseases^19,99,100^. However, the molecular mechanism underlying TDP-43 propagation is still unclear. Thus, further in-depth studies are warranted to fully elucidate whether different propagation mechanisms occur in each of the various cell types in the central nervous system. Most previous studies have focused on the propagation of TDP-43 from neurons to neurons. However, TDP-43 is expressed in many tissues and cell types, including glial cells in the central nervous system. In particular, several studies have revealed that the overexpression of TDP-43 in astrocytes can induce non-cell autonomous neuronal toxicity^101^. Accordingly, the propagation of TDP-43 by glial cells may also have an important role in the progression of neurodegenerative diseases. Therefore, studies on glia-to-neuron TDP-43 propagation and neuron-to-glia TDP-43 propagation should be conducted.

**Table 1:** Experimental in vitro and in vivo models for the TDP-43 propagation.

Method	TDP-43 donor cell/source	Recipient cell	TDP-43 propagation/Seeding activity	Reference
In vitro				
Conditioned media	CM from HEK293 cells expressing TDP-43	HEK293 cells, primary mouse cortical neuron	O	^84^
	CM from HEK293 cells treated with ALS insoluble factions	HEK293 cells	X	^86^
	CM from HEK293 cells expressing wild-type TDP-43 or mutant TDP-43 ΔNLS	HEK293 cells, primary mouse spinal cord cultured cells	X	^87^
Co-culture system	SH-SY5Y cells harboring phosphorylated TDP-43 aggregates	SH-SY5Y cells	O	^85^
	HEK293 cells containing pTDP-43 aggregates	HEK293 cells	O	^86^
	HEK293 cells expressing TDP-43	HEK293 cells	O	^84^
	SH-SY5Y cells expressing HA-tagged TDP-43	SH-SY5Y cells	O	^88^
	NSC-34 cells expressing wild-type TDP-43-tdTomato	NSC-34 cells expressing mutant TDP-43 ^G294A^-tGFP	O	^89^
Using brain lysate or CSF from patients	Insoluble fraction from brain tissue of ALS or FTLD-TDP patient	SH-SY5Y cells	O	^85^
	ALS patient-derived cerebellum or cortex lysate	Mouse primary cortical neuron	Only in case of treatment of cortex lysate	^84^
	Insoluble fraction from CNS tissues of ALS patient	HEK293 cells, NSC-34 cells	O	^86^
	Insoluble fraction from brain tissue of FTLD-TDP patient	HEK293 cells expressed TDP-43	O	^90^
	CSF-derived ALS or FTD patient	U251 cells	O	^91^

Method	Material	Model	Symptoms	Reference
In vivo				
Intraperitoneal injection	Administration of GW4896 (a chemical inhibitor of exosome biogenesis)	Transgenic mice expressing human TDP-43^A315T^ mutant	Exaggerated abnormal behavioral phenotype	^92^
Stereotaxic injection	Stereotaxic injection of extracts derived FTLD-TDP patients	Transgenic mice expressing cytoplasmic human TDP-43 (lines CamKIIa-hTDP-43^NLSm^, rNLS8, and CamKIIa-208)	Time-dependent propagation of TDP-43 pathology	^93^
